# The therapeutic efficacy of intensive medical therapy in ameliorating high-density lipoprotein dysfunction in subjects with type two diabetes

**DOI:** 10.1186/s12944-016-0314-4

**Published:** 2016-08-27

**Authors:** Sangeeta Kashyap, Karim Kheniser, Ling Li, James Bena, Takhar Kasumov

**Affiliations:** 1 Departemnt of Endocrinology and Metabolism, Cleveland Clinic, 9500 Euclid Avenue, Cleveland, OH 44195 USA; 2 Department of Core Facilities, Cleveland Clinic, 9500 Euclid Avenue, Cleveland, OH 44195 USA; 3 Department of Quantitative Health Sciences, Cleveland Clinic, 9500 Euclid Avenue, Cleveland, OH 44195 USA; 4 Department of Hepatology, Cleveland Clinic, 9500 Euclid Avenue, Cleveland, OH 44195 USA; 5 Present address: Department of Pharmaceutical Sciences, Northeast Ohio Medical University, 4209 St. R. 44, PO Box 95, Rootstown, OH 44272 USA

**Keywords:** Intensive medical therapy, Paraoxonase one, Pro-inflammatory high-density lipoproteins, Myeloperoxidase

## Abstract

**Background:**

To determine whether 12 months of intensive medical therapy (IMT) improves HDL functionality parameters in subjects with type II diabetes (T2D).

**Methods:**

Retrospective, randomized, and controlled 12-month IMT intervention trial that enrolled 13-subjects with T2D (age 51- years, fasting glucose 147 mg/dL, body mass index [BMI] 36.5 kg/m^2^) and nine healthy control (46-years, fasting glucose 90 mg/dL, BMI 26.5 kg/m2). Subjects with T2D underwent IMT and HDL functionality measures (pro-inflammatory index of high-density lipoprotein (pHDL)), paraoxonase one (PON1), ceruloplasmin (Cp), and myeloperoxidase (MPO) activity were performed on samples at baseline and at 12-months following IMT.

**Results:**

At baseline, pHDL index was significantly higher in subjects with T2D (*p* < 0.001) and apolipoprotein A-1 levels were significantly lower (*p* = 0.013) vs. controls. After 12-months, there was a trend for improved pHDL activity (*p* = 0.083), as indicated by intent-to-treat analysis, but when the non-adherent subject was omitted (per-protocol), significant attenuations in pHDL activity (*p* = 0.040) were noted; Δ pHDL activity at 12-months was associated with Δ weight (*r* = 0.62, *p* = 0.032) and Δ fasting glucose (*r* = 0.65, *p* = 0.022). Moreover, PON1 activity significantly improved (*p* < 0.001). The aforementioned occurred in association with improvements in inflammatory markers (i.e., C-reactive protein & tumor necrosis factor), hemoglobin A1C, fasting glucose, triglycerides, high-density lipoprotein levels and adipokines.

**Conclusion:**

IMT ameliorates pHDL index and significantly improves anti-oxidative function, as measured by PON1. Improvements in weight and fasting glucose mediated the decrease in pHDL index. Pharmacological aids and lifestyle modification are required to improve cardiovascular risk factors, subsequent mortality risk, and promote T2D remission. Application of either form of therapy alone may only have relatively miniscule effects on the aforementioned factors, in relation to the aggregate.

## Background

Undoubtedly, physical inactivity, mass consumption of calorically dense and vitamin deficient processed foods, and environmental factors have contributed to the unabated rise in obesity and type II diabetes mellitus (T2D). In the ensuing few decades, the prevalence of T2D is expected to rise from 2.8 to 4.4 % [1]. As a result, the incidence of cardiovascular related deaths will increase correspondingly [2]. The heightened prevalence of cardiovascular disease (CVD) and cardiovascular-related deaths among subjects with T2D can be partially explained by reductions in high-density lipoprotein (HDL) levels [3]. On a particle number basis, diminutions in HDL would concomitantly reduce the ability of HDL to exert their physiochemical functions, which include potent anti-atherogenic, anti-oxidative, cholesterol efflux, vasodilatory, and anti-inflammatory functions [4]. Consequently, given their constellation of pertinent functions, improving HDL levels in subjects with T2D has been one of the focal points of medical therapy.

However, although empirical evidence has indicated that HDL levels are negatively correlated with CVD [5], the association may become distorted in subjects with T2D, due to the presence of HDL dysfunction. Indeed, T2D is marked by depressed anti-oxidative function and increased pro-inflammatory HDL activity (pHDL); not only does pHDL precipitate the formation of oxidized LDL, but they are incapable of preventing and inactivating the formation of LDL-derived oxidized phospholipids [6–8]. The aforesaid is precipitated by a milieu that engenders deleterious morphological alterations to HDL, such as promoting the disassociation of apolipoprotein A-1 (apoA-1) from HDL [9]. Specifically, its etiology directly manifests from increased oxidative stress, chronic inflammation, hyperglycemia, and hypertriglyceridemia [4]. The aggregate perturbs HDL function by promoting HDL glycosylation [10], reducing hepatic synthesis of apoA-1 [11], replacing apoA-1 and paraoxonase (PON1) with serum amyloid A (SAA) [12], and post-translation modification (PTM) of apoA-1 [13]; concurrently, HDL enrichment in triglycerides (TG) and the depletion of cholesterol ester in its hydrophobic core, which is a consequent of increased adipose tissue lipolysis, increased hepatic TG synthesis and the aberrant activity of cholesterol ester transfer protein (CETP), lipoprotein lipase (LPL), lecithin cholesterol acyl-transferase (LCAT) and hepatic lipase (HL) are also contributory [14, 15].

As a result, HDL antioxidative, cholesterol efflux, and anti-inflammatory functions become attenuated [4]. Even more alarming is that dysfunctional HDL have been demonstrated to propagate the inflammatory cascade and consequently may play a pivotal role in inducing plaque progression [16]; pHDL are associated with diminished levels of PON1 [6], which would reduce HDL ability to inhibit low-density lipoproteins (LDL) oxidation by prooxidant enzymes such as myeloperoxidase (MPO) [17]. Therefore, assaying HDL function through qualitative means has supplanted quantitative measurements because the latter fails to provide any insight into its true physiochemical functions. Several lines of evidences suggest that pharmacological and/or lifestyle interventions ameliorate HDL dysfunction.

For instance, CETP inhibitors can improve HDL functionality by increasing the ratio of cholesterol esters (CE) to TG [18]. Administration of these drugs with niacin have been demonstrated to reduce apoA-1 fractional catabolic rate in patients with T2D [19]. Further, statins have been noted to attenuate HDL-inflammatory index levels in subjects with coronary heart disease [20]. Correspondingly, physical activity has been demonstrated to improve LCAT, cholesterol efflux, and PON1 activity, collectively conferring potent anti-atherogenic effects [21–24]. Similarly, a three week dietary and physical activity intervention has been noted to significantly improve pHDL activity [25].

However, the aforementioned trials utilized interventions that were of relatively short duration (≤ 3 months) and none analyzed the sustained effects of intensive medical therapy (IMT) (i.e., behavioral and pharmacological treatment) on subjects who are obese and primarily stricken with T2D. Therefore, the purpose of this retrospective study was to ascertain how and to what extent a long-term (one year) IMT intervention would ameliorate HDL dysfunction in obese subjects with T2D; specifically, elucidating if pro-inflammatory HDL and PON1 activity are improved and if they are associated with reductions in MPO, cholesterol, TG, glycaemia, and body mass index (BMI) is most prudent. *The central hypothesis was that hyperglycemia induced oxidative stress in subjects with T2D causes loss of anti-oxidative and anti-inflammatory functions of HDL. Moreover, intensive glycemic control will restore HDL functionality.* Therefore, we assessed the in vitro functionality of HDL in subjects with T2D vs. healthy control subjects at baseline and following twelve months of intensive diabetes treatment with lifestyle counseling and glucose lowering medications.

## Methods

Retrospectively, we conducted a study on subjects with T2D who were enrolled in a randomized, controlled, single center trial and received intensive medical therapy (IMT) for 12 months. Specifically, IMT was denoted as referencing the most novel therapeutic guidelines set forth by the American Diabetes Association, which included pharmacological treatment and lifestyle modification counseling (hypocaloric, carbohydrate controlled diet and moderate physical activity) provided by quarterly visits with study endocrinologists and an annual visit with diabetic educators. Prescribed pharmacological agents included biguanides (BG), incretin mimetics, thiazolidinediones (THZ), sulfonylureas (SF), and insulin analogs. Of which, BG, incretin mimetics, and insulin were the most commonly administered. Inclusion criteria consisted of an age of 20 to 60 years, HGBA1C levels of ≥ 7.0 %, and a BMI of 27 to 43 kg/m^2^. The research was performed in accordance with the Declaration of Helsinki and all subjects provided written informed consent. The trial was approved by the institutional review board at the Cleveland Clinic.

All chemicals were purchased from Sigma-Aldrich Chemical Company (St Louis, MO) except where indicated otherwise. Blood glucose was measured using the glucose oxidase method (Beckman glucose analyzer, Beckman Instruments, Fullerton, CA), and serum insulin levels were measured using a commercial enzyme-linked immunosorbent assay kit (Linco Research, St Charles, MO). Homeostasis model assessment (HOMA) was calculated as a measure of insulin resistance. Adipokines (adiponectin and leptin) were measured using a high-sensitivity human cytokine multiplex kit (LINCOplex; Linco Research, St Charles, MO). Serum C-reactive protein (CRP) concentration was measured with high-sensitivity sandwich enzyme-linked immunosorbent assay. Total cholesterol, HDL-cholesterol, TG, and glycosylated hemoglobin (HGBA1C) were measured by standard methods in the certified clinical laboratory.

### Outcomes

Primary outcome measures were assessed at baseline and one year after intervention, for T2D, while they were assayed at baseline, in controls.

#### Human ApoA-1 quantification

Human ApoA-1 was quantified by immunoassay method on the Abbott ARCHITECT ci8200 Integrated Analyzer System (Abbott Labs, Abbott Park, IL).

#### PON1 activity

PON1 activity in 5 μl serum was assayed based on a fluorescence assay (excitation at 360 nm and emission at 450 nm) using EnzChek (Molecular Probes, Inc. Eugene, OR) Paraoxonase Assay Kit protocol. Paraoxon was used as a substrate.

#### Pro-inflammatory index of HDL

A modification of the cell-free assay developed by Navab and colleagues [8] was used to quantify pro-inflammatory HDL [26]. This assay measures the anti-oxidant capacity of plasma proteins to prevent Cu^2^-induced oxidative stress. Briefly, HDL oxidation in apoB-depleted plasma was initiated with Cu^2+^ and rates of HDL oxidation quantified with 2′,7′-dichlorodihydrofluorescein (DCFH) in a microtiter plate at 37 °C. Fluorescent emission with 530 nm wavelength was measured after serial excitation at 485 nm.

#### MPO activity

The peroxidase activity of MPO in serum was measured by spectrophotometer at 650 nm with 3,3′5,5′-tetramethylbenzidine (TMB) as a substrate.

#### Ceruloplasmin (Cp) activity

The amino oxidase activity of Cp in serum was measured by spectrophotometer at 530 nm with p-phenylenediamine (based on the article by Wei et al. [27], with modifications based on ref. Lehmann et al. [28]).

### Statistical analysis

Analysis was based on an intention-to-treat and per-protocol. Due to nonparametric data, continuous variables were summarized with medians and quartiles, whilst categorical factors were summarized via frequency and percentiles. To assess group differences, a Fisher’s exact test was used, with respect to categorical variables. Independent group differences for continuous variables were assessed using the Wilcoxon rank sum test, whereas time-related within-group differences for continuous variables were ascertained via the Wilcoxon signed rank test. Lastly, spearman correlation was used to deduce associations between parameters. Data was analyzed via SAS software (version 9.3; Cary, NC).

## Results

### Study cohort and baseline characteristics

Thirteen subjects with T2D were identified as subjects who met the inclusion criteria, while nine subjects served as the control. Table 1 depicts baseline differences between subjects with T2D and controls. Although BMI was significantly greater among subjects with T2D (*p* < 0.001), there were no differences in demographic variables between controls (*n* = 7 [77.8 %] females, age 46 [30.0, 57.2] years, BMI 25.6 [21.2, 26.9] kg/m^2^) and subjects with T2D (*n* = 8 [61.5 %] females, age 51.1 [43.7, 55.3] years, BMI 36.5 [33.4, 37.5] kg/m^2^). For primary outcome measures, pHDL activity was significantly higher (0.49 [0.42, 0.54] vs. 0.26 [0.25, 0.34], *p* < 0.001) and apoA-1 levels were significantly lower (124.5 [111.4, 128.7] vs. 144.2 [129.4, 174.5], *p* = 0.013), while there were non-significant differences in PON1 (0.01 [0.01, 0.01] vs. 0.01 [0.01, 0.01], *p* = 0.15), Cp (0.16 [0.14, 0.17] vs. 0.18 [0.14, 0.25], *p* = 0.22), and MPO activity (6.5 [5.6, 20.9] vs. 11.8 [4.4, 14.6], *p* = 0.31). With respect to metabolic parameters, HDL-cholesterol (41.0 [37.0, 45.0] vs. 63.0 [45.0, 72.0], *p* = 0.005), fasting insulin ([INS-0], 32.4 [16.9, 41.5] vs. 5.9 [5.4, 7.1], *p* < 0.001), fasting glucose ([GLUC], 180.0 [161.0, 240.0] vs. 90.0 [83.0, 93.0], *p* < 0.001), HOMA (14.4 [6.8, 22.0] vs. 1.2 [1.1, 1,5], *p* < 0.001) were potentiated, whilst TG (174.0 [152.0, 337.0] vs. 74.0 [50.0, 86.0], *p* = 0.008) concentrations were attenuated among T2D; non-significant differences were observed in total cholesterol (187.0 [172.0, 202.0] vs. 175.0 [143.0, 195.0], *p* = 0.32) and LDL-cholesterol (104.0 [82.0, 114.0] vs. 85.0 [65.5, 95.5], *p* = 0.15). Finally, systolic blood pressure ([SBP], 137.0 [133.0, 156.0] vs. 108.0 [104.0, 115.0], *p* < 0.001), and diastolic blood pressure ([DBP], 89.0 [83.0, 91.0] vs. 66.0 [65.0, 69.0], *p* < 0.001) were significantly greater among T2D.

**Table 1:** Differences in baseline characteristics between subjects with T2D and controls

	Overall (*n* = 22)		Control (*n* = 9)		D2M (*n* = 13)		
Parameter	*n*	Summary	*n*	Summary	*n*	Summary	*p*-value
Fasting glucose (mg/dl)	22	147.0 [91.0,198.0]	9	90.0 [83.0,93.0]	13	180.0 [161.0,240.0]	***< 0.001*** ^***b***^
BMI (kg/m^2^)	22	31.9 [26.4,37.0]	9	25.6 [21.2,26.9]	13	36.5 [33.4,37.5]	***< 0.001*** ^***b***^
HDL-cholesterol (mg/dl)	22	44.5 [37.0,56.0]	9	63.0 [45.0,72.0]	13	41.0 [37.0,45.0]	***0.005*** ^***b***^
Triglycerides (mg/dl)	22	141.5 [82.0,187.0]	9	74.0 [50.0,86.0]	13	174.0 [152.0,337.0]	***0.008*** ^***b***^
HOMA	22	4.8 [1.3,14.8]	9	1.2 [1.1,1.5]	13	14.4 [6.8,22.0]	***< 0.001*** ^***b***^
Total cholesterol (mg/dl)	22	180.0 [158.0,202.0]	9	175.0 [143.0,195.0]	13	187.0 [172.0,202.0]	0.32^b^
LDL-cholesterol (mg/dl)	21	95.0 [79.0,109.0]	8	85.0 [65.5,95.5]	13	104.0 [82.0,114.0]	0.15^b^
Fasting insulin (mU/l)	22	10.6 [6.3,34.3]	9	5.9 [5.4,7.1]	13	32.4 [16.9,41.5]	***<0.001*** ^***b***^
Age (years)	22	49.0 [41.7,55.6]	9	46.0 [30.0,57.2]	13	51.1 [43.7,55.3]	0.37^b^
Female	22	15 (68.2)	9	7 (77.8)	13	8 (61.5)	0.65^d^
Systolic blood pressure (mmHG)	22	126.0 [115.0,138.0]	9	108.0 [104.0,115.0]	13	137.0 [133.0,156.0]	***< 0.001*** ^***b***^
Diastolic blood pressure (mmHG)	22	76.0 [68.0,89.0]	9	66.0 [65.0,69.0]	13	89.0 [83.0,91.0]	***< 0.001*** ^***b***^
MPO activity (mOD/μL/min)	21	8.3 [5.1,14.8]	9	11.8 [4.4,14.6]	12	6.5 [5.6,20.9]	0.31^b^
pHDL assay (RFU mg HDLc/min)	21	0.42 [0.28,0.50]	9	0.26 [0.25,0.34]	12	0.49 [0.42,0.54]	***< 0.001*** ^***b***^
Cp activity (μmol.min-1/mL)	21	0.16 [0.14,0.18]	9	0.18 [0.14,0.25]	12	0.16 [0.14,0.17]	0.22^b^
PONI1 activity (nmole.min-1/μL)	21	0.01 [0.01,0.01]	9	0.01 [0.01,0.01]	12	0.01 [0.01,0.01]	0.15^b^
apoA-1 (mg/dl)	20	128.6 [114.8,143.7]	9	144.2 [129.4,174.5]	11	124.5 [111.4,128.7]	***0.013*** ^***b***^

Intent-to-treat analysis. Values presented as median [P25, P75] or *n* (column %)

*p*-values: b = Wilcoxon rank sum test, d = Fisher’s exact test

### Efficacy of IMT

**Fig. 1:**
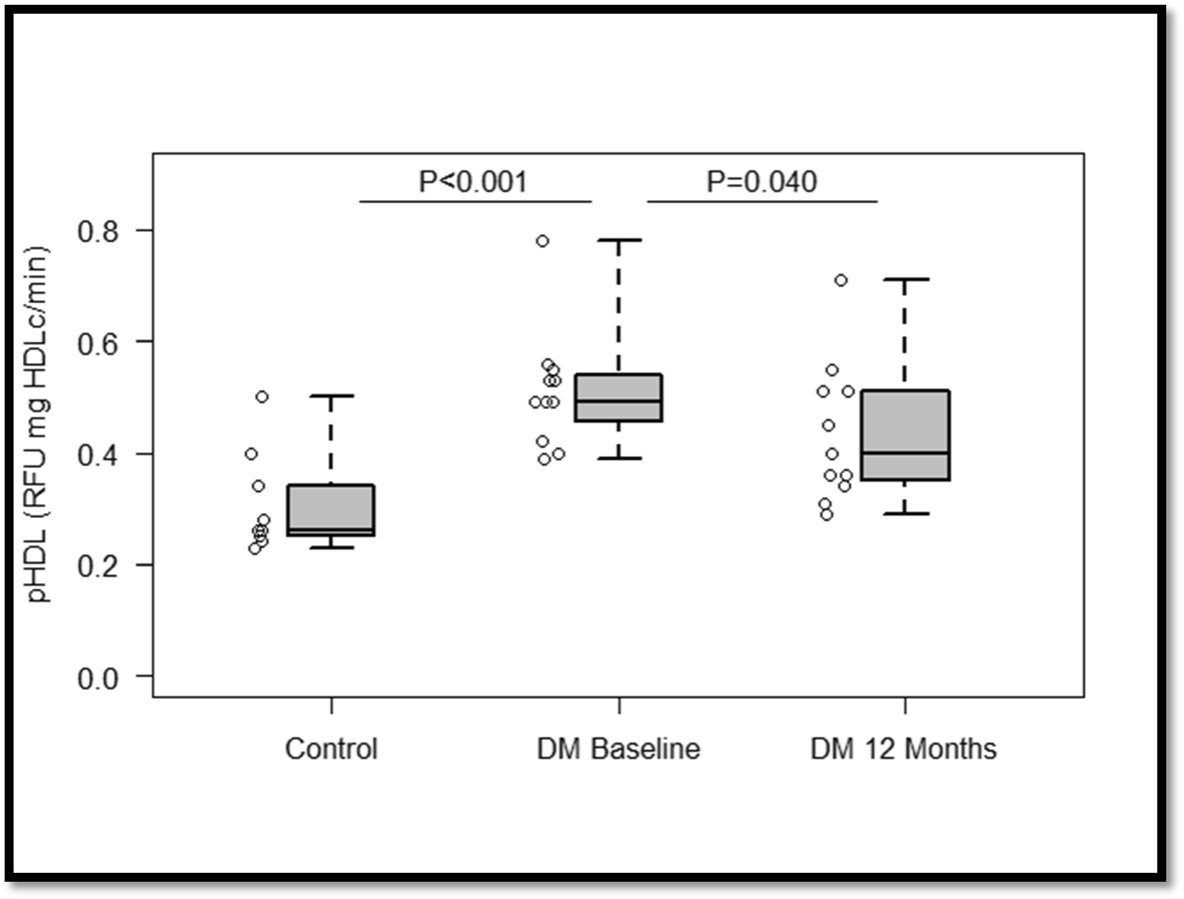
Box plot depicting differences in pHDL activity at baseline and after 12 months of IMT. As illustrated, there were significant differences at baseline between subjects with T2D and controls. After 12 months of IMT, a per-protocol analysis, which omitted the non-adherent subject, indicated that pHDL activity significantly decreased. *p*-values: Wilcoxon rank sum test for intergroup differences, whereas within-group differences were analyzed via the Wilcoxon signed rank test. DM, type II diabetics; pHDL, pro-inflammatory high-density lipoprotein activity

Table 2 shows the effects of 12 months of IMT on primary outcome measures, metabolic parameters, anthropometric characteristics, inflammatory markers, and endocrine parameters in subjects with T2D, in relation to baseline. For primary outcome measures, there was a trend for reduced pHDL activity (0.43 [0.35, 0.51] vs. 0.49 [0.42, 0.54], *p* < 0.083), but when subjected to a per-protocol analysis the *p*-value became significant (0.40 [0.34,0.51] vs. 0.49 [0.42,0.54], *p* = 0.040) (Fig. 1); further, significant increases in PON1 activity (0.02 [0.01, 0.03] vs. 0.01 [0.01, 0.01], *p* < 0.001), whereas MPO (5.9 [4.9, 8.6] vs. 6.5 [5.6, 20.9], *p* = 0.18) and Cp (0.14 [0.13, 0.19] vs. 0.16 [0.14, 0.17], *p* = 0.98] were not significantly different. As it pertains to metabolic variables, HDL-cholesterol (50.0 [46.0, 56.0] vs. 41.0 [37.0, 45.0], *p* = <0.001), GLUC (108.0 [88.0, 128.0] vs. 180.0 [161.0, 240.0], *p* = 0.002), HGBA1C (6.1 [6.0, 6.7] vs. 9.1 [8.9, 10.2], *p* < 0.001), and TG (119.0 [94.0, 152.0] vs. 174.0 [152.0, 337.0], *p* < 0.001) significantly improved, while total cholesterol (182.0 [152.0, 209.0] vs. 187.0 [172.0, 202.0], *p* = 0.53) and LDL-cholesterol (91.0 [73.0, 127.0] vs. 104.0 [82.0, 114.0], *p* = 0.58) were not significantly different. Moreover, IMT induced significant decreases in BMI (28.0 [26.4, 30.7] vs. 36.5 [33.4, 37.5], *p* < 0.001) and weight (83.5 [69.4, 92.1] vs. 96.8 [93.2, 111.1], *p* < 0.001), while congruently reducing inflammatory markers such as CRP (1.00 [0.50, 3.1] vs. 5.5 [3.1, 6.2], *p* < 0.001) and tumor necrosis factor ([TNF] 1.04 [0.61, 1.1] vs. 1.4 [1.02, 2.0], *p* < 0.006). Lastly, adiponectin levels increased (6.1 [4.7, 9.2] vs. 2.8 [2.4, 4.0], *p* < 0.001), whilst leptin concentrations decreased (13.6 [9.3, 23.6] vs. 22.7 [15.8, 32.4], *p* = 0.006).

**Table 2:** Differences in clinical characteristics in subjects with T2D after twelve-months of IMT

	Baseline (*n* = 13)		Follow-up (*n* = 13)		Change (*n* = 13)		
Parameter	*n*	Summary	*n*	Summary	*n*	Summary	*p*-value
Fasting glucose (mg/dl)	13	180.0 [161.0,240.0]	13	108.0 [88.0,128.0]	13	−87.0 [−127.0,−41.0]	***0.002***
HGBA1C (%)	13	9.1 [8.9,10.2]	13	6.1 [6.0,6.7]	13	−3.0 [−4.1,−2.1]	***< 0.001***
Body weight (kg)	13	96.8 [93.2,111.1]	13	83.5 [69.4,92.1]	13	−14.7 [−37.9,−5.9]	***< 0.001***
BMI (kg/m^2^)	13	36.5 [33.4,37.5]	13	28.0 [26.4,30.7]	13	−5.9 [−9.2,−2.0]	***< 0.001***
HDL-cholesterol (mg/dl)	13	41.0 [37.0,45.0]	13	50.0 [46.0,56.0]	13	7.0 [4.0,16.0]	***< 0.001***
Triglycerides (mg/dl)	13	174.0 [152.0,337.0]	13	119.0 [94.0,152.0]	13	−42.0 [−135.0,−13.0]	***< 0.001***
Tumor necrosis factor	13	1.4 [1.02,2.0]	13	1.04 [0.61,1.1]	13	−0.39 [−0.68,−0.23]	***0.006***
C-reactive protein (mg/dl)	13	5.5 [3.1,6.2]	13	1.00 [0.50,3.1]	13	−2.5 [−4.5,−1.7]	***< 0.001***
Adiponectin (ug/ml)	13	2.8 [2.4,4.0]	13	6.1 [4.7,9.2]	13	2.9 [2.2,5.5]	***0.001***
Leptin (ng/ml)	13	22.7 [15.8,32.4]	13	13.6 [9.3,23.6]	13	−11.5 [−16.6,−2.0]	***0.006***
Total cholesterol (mg/dl)	13	187.0 [172.0,202.0]	13	182.0 [152.0,209.0]	13	2.0 [−38.0,13.0]	0.53
LDL-cholesterol (mg/dl)	13	104.0 [82.0,114.0]	13	91.0 [73.0,127.0]	13	−2.0 [−18.0,11.0]	0.58
MPO activity (mOD/μL/min)	12	6.5 [5.6,20.9]	12	5.9 [4.9,8.6]	12	−1.3 [−9.0,0.30]	0.18
pHDL assay (RFU mg HDLc/min)	12	0.49 [0.42,0.54]	12	0.43 [0.35,0.51]	12	−0.11 [−0.13,0.01]	0.083
pHDL assay (RFU mg HDLc/min)	11	0.49 [0.42,0.54]	11	0.40 [0.34,0.51]	11	−0.13 [−0.13, −0.02]	***0.040*** ^***a***^
Cp activity (μmol.min-1/mL)	12	0.16 [0.14,0.17]	12	0.14 [0.13,0.19]	12	0.00 [−0.02,0.02]	0.98
PONI activity (nmole.min-1/μL)	12	0.01 [0.01,0.01]	12	0.02 [0.01,0.03]	12	0.01 [0.00,0.02]	***< 0.001***
Systolic blood pressure (mmHG)	13	137.0 [133.0,156.0]	13	129.0 [117.0,141.0]	13	−8.0 [−22.0,3.0]	0.051
Diastolic blood pressure (mmHG)	13	89.0 [83.0,91.0]	13	79.0 [72.0,85.0]	13	−8.0 [−11.0,−1.00]	***0.005***

Values presented as median [P25, P75]

*p*-values: Wilcoxon signed rank test. a = per-protocol analysis

### Association between primary HDL functionality outcome variables and clinical/metabolic parameters

**Fig. 2:**
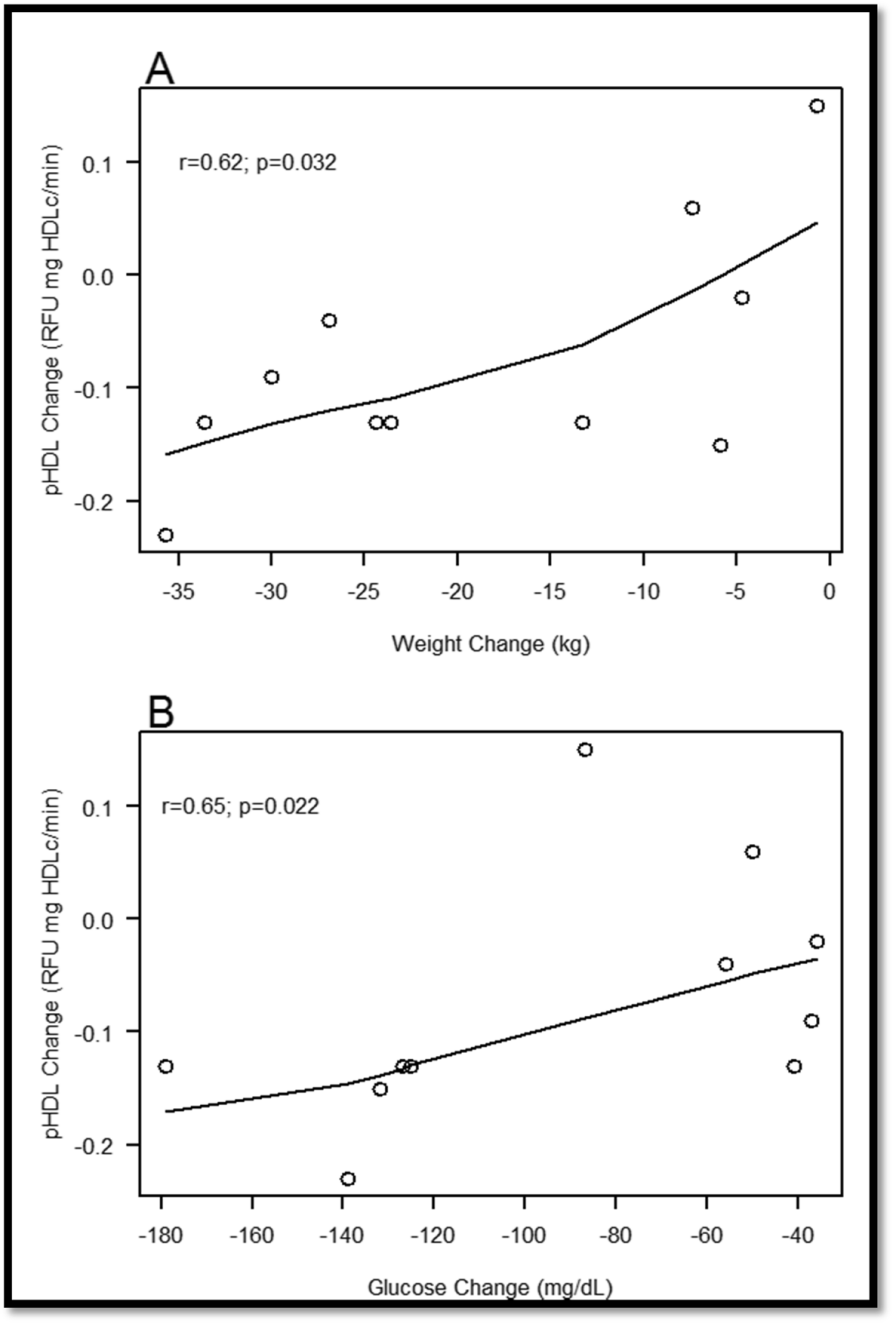
**a** Positive correlation between Δ pHDL activity and Δ weight in subjects with T2D, after 12 months of IMT. **b** Positive association between Δ pHDL activity Δ fasting glucose in subjects with T2D, after 12 months of IMT. The figure highlights that large decreases in weight or glucose were related with heightened decrements in pHDL activity. Intent-to-treat analysis; *r*-value: Spearman correlation. pHDL, pro-inflammatory high-density lipoproteins

Table 3 illustrates relations between primary outcome variables and clinical marker at baseline and after 12 months of IMT for subjects with T2D and among the aggregate of both groups. Baseline combined group correlations indicated that pHDL was positively correlated with BMI (*r* = 0.74, *p* < 0.001, 95 % confidence interval [CI] 0.42–1.00), INS-0 (*r* = 0.73, *p* < 0.001, CI 0.41–1.00), GLUC (*r* = 0.71, *p* < 0.001, CI 0.37–1.00), HOMA (*r* = 0.72, *p* < 0.001, CI 0.38–1.00), and TG (*r* = 0.53, *p* = 0.014, CI 0.12–0.94), whereas it was negatively associated with HDL-cholesterol levels (*r* = −0.65, *p* = 0.001, CI ^−^1.00–^−^0.29). Moreover, MPO and PON1 activity were positively correlated with total cholesterol (*r* = 0.55, *p* = 0.011, CI 0.14–0.95; *r* = 0.46, *p* = 0.036, CI 0.03–0.89, respectively), whereas Cp activity was negatively associated with age (*r* = −0.46, *p* = 0.037, CI ^−^0.88–^−^0.03). Conversely, with respect to subjects with T2D at baseline, PON1 and Cp activity were correlated with HDL-cholesterol (*r* = 0.78, *p* = 0.003, CI 0.35–1.00) and CRP (*r* = 0.66, *p* = 0.019, CI 0.13–1.00), respectively; further, pHDL was significantly associated with LDL-cholesterol (*r* = 0.59, *p* = 0.042, CI 0.03–1.00). After 12 months of IMT, the difference in (Δ) Cp activity was significantly associated with Δ CRP (*r* = 0.69, *p* = 0.012, CI 0.19–1.00) and Δ TNF (*r* = 0.79, *p* = 0.002, CI 0.36–1.00), while Δ MPO was correlated with Δ total cholesterol levels (*r* = 0.59, *p* = 0.044, CI 0.02–1.00); furthermore, Δ pro-inflammatory index of HDL was related with Δ weight and Δ glucose (*r* = 0.62, *p* = 0.032, CI 0.07–1.00; *r* = 0.65, *p* = 0.022, CI 0.12–1.00, respectively) (Fig. 2).

**Table 3:** Correlations between primary outcome variables and clinical characteristics at baseline and twelve-months

	Parameter	*n*	rho	95 % CI	*p* value
Baseline Combined Group Correlations					
Cp Activity	Age	21	−0.46	(−0.88,−0.03)	***0.037***
MPO Activity	Total cholesterol	21	0.55	(0.14,0.95)	0.011
PON1 Activity	Total cholesterol	21	0.46	(0.03,0.89)	0.036
	Systolic blood pressure	21	0.45	(0.03,0.88)	0.038
pHDL Assay	Body mass index	21	0.74	(0.42,1.00)	< 0.001
	Fasting insulin	21	0.73	(0.41,1.00)	< 0.001
	Fasting glucose	21	0.71	(0.37,1.00)	***< 0.001***
	HOMA	21	0.72	(0.38,1.00)	< 0.001
	High-density lipoproteins	21	−0.65	(−1.00,−0.29)	0.001
	Triglycerides	21	0.53	(0.12,0.94)	0.014
	Systolic blood pressure	21	0.74	(0.41,1.00)	< 0.001
	Diastolic blood pressure	21	0.61	(0.22,0.99)	0.004
Baseline Correlations among T2D					
Cp Activity	C-reactive protein	12	0.66	(0.13,1.00)	0.019
PON1 Activity	High-density lipoproteins	12	0.78	(0.35,1.00)	0.003
pHDL Assay	Low-density lipoproteins	12	0.59	(0.03,1.00)	0.042
One Year Correlations among T2D					
Cp - Difference	C-reactive protein – difference	12	0.69	(0.19,1.00)	0.012
	Tumor necrosis factor – difference	12	0.79	(0.36,1.00)	0.002
MPO - Difference	Fasting cholesterol – difference	12	0.59	(0.02,1.00)	0.044
pHDL - Difference	Weight – difference	12	0.62	(0.07,1.00)	0.032
	Fasting glucose – difference	12	0.65	(0.12,1.00)	0.022

Intent-to-treat analysis, *r*-values: Spearman correlation

*Cp* ceruloplasmin, *MPO* myeloperoxidase, *PON1* paraoxonase one, *pHDL* pro-inflammatory high-density lipoproteins

## Discussion

While others have noted the presence of pHDL activity and defective HDL anti-oxidative function in subjects with T2D [6, 7], the principal and novel outcome was that pHDL activity may be ameliorated with IMT. Particularly, although there was a two-fold difference in pHDL activity at baseline between controls and subjects with T2D, HDL dysfunction was ameliorated, such that there was a trend for reduced pHDL activity after 12 months of IMT. However, when subjected to a per-protocol analysis, which removed the non-adherent subject, the results became modestly significant (*p* = 0.040). In particular, IMT fostered reductions in pHDL activity after 12 months, in nine of the twelve type II diabetic subjects, in which it was measured, whereas it was noted to increase in three. Along with pHDL activity, one subject’s metabolic profile was exacerbated at 12 months, thereby demonstrating that the subject was non-adherent to therapy; while pHDL activity was refractory, even in the midst of decreases in blood glucose and TG, in the remaining two subjects. Further, in the aforementioned non-responders, weight was modestly reduced or maintained at 12 months. Consequently, attenuations in pHDL activity may necessitate concomitant significant decreases in blood glucose and weight. Reductions in GLUC would attenuate HDL glycosylation and oxidation, which would improve HDL functionality [10].

As such, correlational analysis indicated that Δ blood glucose and Δ weight were significantly correlated with Δ pHDL activity at 12 months, which affirmed the hypothesis that there would be a relationship between glycaemia and pHDL activity. Of note, the results from this study advance the knowledge on the effectiveness of dietary and physical activity paradigms that foster weight-loss by demonstrating that additional benefits (i.e., improved HDL function, etc.) can be accrued from these modalities. Similarly, pharmacotherapy that attenuates blood glucose levels may provide additional benefits outside of the effects on GLUC and insulin control; this has been demonstrated with CETP inhibitors, statins, and niacin medications [18–20, 29]. Consequently, the synergistic actions and thus the aggregate of physical activity, pharmacotherapy, and dietary adherence would provide the most benefit than one modality of IMT alone, given that HDL dysfunction is caused by an overabundance of factors.

Baseline combined group analysis demonstrated that pHDL activity was related with BMI, INS-0, blood glucose, HOMA, HDL-cholesterol, and TG, while it was associated with LDL-cholesterol in subjects with T2D, when diabetes was uncontrolled. Therefore, pHDL activity may be associated with divergent parameters, depending on the population and severity of disease, and a myriad of variables may precipitate HDL dysfunction.

The association between PON1 and HDL at baseline for T2D underpins them as mutualistic beneficiaries, but this correlation is more frequently noted among controls [30]. Interestingly, the association was not evident at one year, which suggests that other factors mediate their association [30]. Noteworthy, HDL antioxidant activity as assayed by PON1 was not different between subjects with T2D and controls at baseline, which has been corroborated in other trials [20, 31, 32]. The results indicate that PON1 activity may be resilient to the hyperglycemic, hypertriglyceridemic, and pro-inflammatory milieu that is present in subjects with T2D; further, PON1 activity may only be significantly reduced in subjects with long-standing T2D. In addition, the non-significant intergroup differences in PON1 activity and the heightened levels of pHDL activity at baseline attests that other factors mediate HDL anti-inflammatory dysfunction. Indeed, Navab et al. [33] observed that PON1 activity alone did not explain the divergences in HDL function. Further, PAF activity is concomitantly diminished [6]. Therefore, decrement activity in the aforesaid parameter may have also been causal to HDL dysfunction. However, PON1 activity improved after 12 months of IMT, which indicates that it may be more amenable to IMT, given that it improved significantly, while there was a trend for improved pHDL activity. Potentiated PON1 activity would heighten HDL ability to inhibit LDL oxidation and mildly oxidized LDL, which results in reduced monocyte chemotaxis [8, 16, 33], and assisting in inhibiting the formation of the one of the prime initial catalysts to the development of atheroma: oxidized LDL [34]. Further, HDL is less prone to glucose-induced lipid peroxidation when PON1 activity is elevated [10].

Albeit non-significant, Cp and MPO activity were lower at baseline and remained so even after 12 months of IMT, relative to controls. Interestingly, the aforementioned did not significantly decrease, while inflammatory markers (i.e., CRP and TNF) decreased significantly in subjects with T2D. Inflammation is requisite to increased acute phase protein levels [4], and improvements in PON1 activity would expectedly attenuate MPO activity, but this was not significant; MPO and PON1 reciprocate each others actions [35]. Further, acute phase proteins (e.g., Cp) have been demonstrated to replace PON1, LCAT, and PAF, which precipitates pHDL activity [8, 16]. Cp has also been noted to facilitate LDL oxidation [36]. Moreover, MPO activity can negatively modulate HDL function by impairing its antiapototic and anti-inflammatory capacity, while congruently increasing its pro-inflammatory activity [37]. Similarly, MPO intermediates (e.g., hypothiocyanous and hypochlorous acid) induce apoA-1 oxidation, which would impair its cholesterol efflux capacity and consequently its anti-atherogenic functions [13, 38]. The former selectively targets tryptophan residues [13]. However, even though elevated levels of Cp and MPO activity may cause HDL dysfunction and contribute to pHDL activity, the results provide credence to the fact that it is unlikely that they had a significant effect on pHDL activity. Indeed, pHDL activity improved irrespectively of significant reductions in Cp and MPO and, relative to controls, their activity was lower at baseline, when pHDL activity was elevated.

Conversely, apoA-1 levels were significantly attenuated among subjects with T2D at baseline [39]. Indeed, subjects with T2D displayed elevated levels of GLUC, TG, and inflammatory markers. Due to the hypertriglyceridemic and hyperglycemic milieu, HDL become enriched in TG and apoA-1 becomes glycosylated [4]. The decreased CE/TG ratio engenders apoA-1 instability and alters the conformation of the central and C-terminal domains [40]. Further, due to chronic inflammation and oxidative stress, apoA-1 is replaced by SAA and undergoes PTM [4]. The aggregate causes reductions in apoA-1 levels and perturbs apoA-1 functionality. Subsequently, their ability to promote cholesterol efflux, remove seeding molecules (i.e., formation of metabolites of linoleic and arachidonic acid by endothelial cells such as 12-lipoxygenase) from LDL, and inhibit LDL oxidation will be abrogated [33, 41, 42]. With respect to the latter, apoA-1 confers antioxidative functions directly, as mentioned, and indirectly via improvements in PON1 activity [42]. Of importance, cholesterol efflux capacity is inversely related with CVD [43], and oxidized LDL promote monocyte adherence and chemotaxis [33]. However, it is plausible that apoA-1 levels increased, given that HDL levels improved after 12 months of IMT [44].

Weight-loss, PON1 activity, and pharmacological aids appreciate HDL levels [9, 30, 45]. Further, it may have been a consequent of reductions in CETP activity, increased hepatic synthesis and lipidation of apoA-1, and increased cholesterol efflux [4]. In regard to CETP inhibitors, torcetrapib, dalcetrapib and evacetrapib have been discontinued either due to adverse effects in the case of the former or modest effects on cardiovascular outcomes in the latter two medications; the effects of novel CETP inhibitors on cardiovascular outcomes is yet to be elucidated [46]. Concurrently, physical activity has been documented to improve cholesterol efflux and LCAT activity, which would increase the ratio of CE to TG [21–24]; thereby, improving HDL stability [40]. Moreover, the significant reductions in TG would favor up-regulated levels of CE, relative to TG in HDL hydrophobic core [4]. Therefore, on a particle number basis, increased HDL concentrations improves their ability to exert their critical physiochemical functions.

## Conclusion

While discerning between the effects of a multidimensional therapeutic paradigm of IMT on HDL function represented a core limitation to the study, the trial sample size was also finite (*n* = 13). However, rather than strictly looking at the acute effects of pharmacotherapy and lifestyle modification on HDL dysfunction, the present study gleaned additional insight into the long-term benefits and efficacy of IMT. To our knowledge, we are first to report the effects of IMT on HDL function during a long-term trial and particularly in obese subjects with T2D. Specifically, we noted that IMT is a feasible approach to mitigate and possibly ameliorate the deleterious effects of T2D. After all, retarding the progression of T2D requires the synergistic application of lifestyle modification and pharmacotherapy, which more aptly treats the disease.

## Abbreviations

apoA-1: Apolipoprotein A-1
BG: Biguanides
BMI: Body mass index
CE: Cholesterol esters
CETP: Cholesterol ester transfer protein
CP: Ceruloplasmin
CRP: C-reactive protein
DBP: Diastolic blood pressure
DCFH: 2′,7′-dichlorodihydrofluorescein
GLUC: Fasting blood glucose
HDL: High-density lipoprotein
HGBA1C: Glycosylated hemoglobin
HL: Hepatic lipase
HOMA: Homeostasis model assessment
IMT: Intensive medical therapy
LCAT: Lecithin cholesterol acyl-transferase
LDL: Low-density lipoproteins
LPL: Lipoprotein lipase
MPO: Myeloperoxidase
MS: Metabolic syndrome
PAF: Platelet-activity factor-acetyl hydrolase
pHDL: Pro-inflammatory high-density lipoproteins
PON1: Paraoxonase one
PTM: Post-translation modification
SAA: Serum amyloid A
SBP: Systolic blood pressure
SF: Sulfonylureas
T2D: Type II diabetes
TG: Triglycerides
THZ: Thiazolidinediones
TNF: Tumor necrosis factor
