# Erratum to: The therapeutic efficacy of intensive medical therapy in ameliorating high-density lipoprotein dysfunction in subjects with type two diabetes

**DOI:** 10.1186/s12944-016-0362-9

**Published:** 2016-11-09

**Authors:** Sangeeta Kashyap, Karim Kheniser, Ling Li, James Bena, Takhar Kasumov

**Affiliations:** 1 Departemnt of Endocrinology and Metabolism, Cleveland Clinic, 9500 Euclid Avenue, Cleveland, OH 44195 USA; 2 Department of Core Facilities, Cleveland Clinic, 9500 Euclid Avenue, Cleveland, OH 44195 USA; 3 Department of Quantitative Health Sciences, Cleveland Clinic, 9500 Euclid Avenue, Cleveland, OH 44195 USA; 4 Department of Hepatology, Cleveland Clinic, 9500 Euclid Avenue, Cleveland, OH 44195 USA; 5 Present address: Department of Pharmaceutical Sciences, Northeast Ohio Medical University, 4209 St. R. 44, PO Box 95, Rootstown, OH 44272 USA

## Erratum

After publication of the original article [1], it came to the authors’ attention that a source of funding was not disclosed in the Declarations section of the paper. The study was in part supported by the American Diabetes Association (1-15-IN-31). The Funding section of the original article should have acknowledged this grant.
